# Classifying Multi-Level Stress Responses From Brain Cortical EEG in Nurses and Non-Health Professionals Using Machine Learning Auto Encoder

**DOI:** 10.1109/JTEHM.2021.3077760

**Published:** 2021-05-05

**Authors:** Ashlesha Akella, Avinash Kumar Singh, Daniel Leong, Sara Lal, Phillip Newton, Roderick Clifton-Bligh, Craig Steven Mclachlan, Sylvia Maria Gustin, Shamona Maharaj, Ty Lees, Zehong Cao, Chin-Teng Lin

**Affiliations:** 1 FEIT, School of Computer ScienceAustralian Artificial Intelligence Institute, University of Technology Sydney 1994 Ultimo NSW 2007 Australia; 2 Neuroscience Research Unit, School of Life SciencesUniversity of Technology Sydney 1994 Ultimo NSW 2007 Australia; 3 Centre for Cardiovascular and Chronic CareUniversity of Technology Sydney 1994 Ultimo NSW 2007 Australia; 4 Department of EndocrinologyRoyal North Shore HospitalThe University of Sydney 4334 Sydney NSW 2006 Australia; 5 Centre for Healthy Futures, Health VerticalTorrens University Australia, Pyrmont Campus 386703 Pyrmont NSW 2009 Australia; 6 Neuroscience Research Australia 6803 Randwick NSW 2031 Australia; 7 School of PsychologyUniversity of New South Wales 7800 Sydney NSW 2052 Australia; 8 Edna Bennett Pierce Prevention Research CenterPennsylvania State University 8082 State College PA 16801 USA; 9 Information and Communication Technology (ICT)University of Tasmania 3925 Hobart TAS 7005 Australia

**Keywords:** Autoencoder, support vector machine, stress classification, electroencephalogram

## Abstract

Objective: Mental stress is a major problem in our society and has become an area of interest for many psychiatric researchers. One primary research focus area is the identification of bio-markers that not only identify stress but also predict the conditions (or tasks) that cause stress. Electroencephalograms (EEGs) have been used for a long time to study and identify bio-markers. While these bio-markers have successfully predicted stress in EEG studies for binary conditions, their performance is suboptimal for multiple conditions of stress. Methods: To overcome this challenge, we propose using latent based representations of the bio-markers, which have been shown to significantly improve EEG performance compared to traditional bio-markers alone. We evaluated three commonly used EEG based bio-markers for stress, the brain load index (BLI), the spectral power values of EEG frequency bands (alpha, beta and theta), and the relative gamma (RG), with their respective latent representations using four commonly used classifiers. Results: The results show that spectral power value based bio-markers had a high performance with an accuracy of 83%, while the respective latent representations had an accuracy of 91%.

## I. Introduction

Mentally, stress can increase anxiety and depression levels as a result of emotional dysregulation [1], [2]. Stress is also associated with a alterations in a person’s cognitive abilities, affecting the memory and attention systems. However, stress affects each individual in a different manner, with some people having a higher tolerance for stress, while other’s tolerance is low. Because of the potential medical risks from stress, studies on the physiological states induced by stress have increased [3]. To induce stress in the laboratory, the Trier Social Stress Test (TSST) [4] and mental arithmetic stress test [5] are commonly used. Once stress is induced, physiological measurements, such as heart rate variability (HRV) [6] and Galvanic Skin Response (GSR) [7] in conjunction with electroencephalograms (EEGs) can be used to study and measure the physiological changes/responses induced by stress. These changes are often correlated with psychological instruments, for example, the Perceived Stress Scale (PSS) [8].

Among multiple physiological measurements, EEG is a widely used non-invasive method due to its excellent temporal resolution and low cost. EEG can also explain more about the underlying brain dynamics which is not attainable with other physiological recordings, such as galvanic skin response (GSR), photoplethysmography signals (PPG) [9] and electrocardiogram (ECG) [10]. Lately, EEG headsets are also growing to be commercially available for recording brain activity in an easy to wear fashion [11], [12]. Benefits such as portability and wearability of EEG devices have encouraged research towards the core applications of this technology. While EEG signals are popular for extracting prominent bio-markers such as P300 [13], steady-state visual evoked potential (SSVEP) [14]. However, it remains a challenge to extract concealed bio-markers such as stress from EEG signals with substantial accuracy. This motivated us towards the study of classifying multi-class stress responses from brain cortical EEG signal.

Recently, extensive research has focused on the identification of stress using EEG- based biomarkers that not only identify stress but also can predict the conditions (or tasks) that cause stress. Multiple studies that focused on how to assess stress-induced in a laboratory or controlled environment used the relative gamma (RG) as a stress bio marker [15]–[18]. There was some studies that used a EEG-based $theta Fz/alpha Pz$ ratio, called the brain load index (BLI) [19], [20] to detect the stress states. Other features, such as frontal asymmetry [21] and coherence [22], have also been used in EEG studies to detect stress. A few EEG studies utilized machine learning methods, such as linear discriminant analysis (LDA) [15], support vector machine (SVM) [23], k-means clustering [24], and k-nearest neighbors (KNN) [25] to detect stress. Some EEG studies showed a classification performance of up to 90% on a two-level classification [23], [26]. The study conducted by [23] showed results with an accuracy of 96% from a two-level classification (resting vs arithmetic test) using SVM, with a sliding window of two seconds and a one-second overlap. While this is an interesting outcome, studies that classify more than two classes are challenging to undertake.

Decreasing the signal-to-noise-ratio in an EEG signal is one of the most main challenges for an EEG data analysis. This could be one of the reasons limiting the capability of classifying more than two levels of a lab induced stress task. This research aims to classify the multiple conditions caused by different lab-induced stress tasks. In this research, we recorded the EEG data of 30 clinically active nurses and 50 non-health professionals while performing 3 different stress-inducing tasks: 1. preparing for a speech 2. delivering a speech and 3. performing an arithmetic task. Machine learning algorithms such as deep neural networks [27], [28], convolution neural networks [29], [30] and ensemble convolution neural networks [31]. Among which, auto-encoder neural network have been shown to learn the underlying representation of the given data by reducing the dimensionality of the data [27], [32]–[34]. After training in an unsupervised technique, auto encoders have been shown to ignore the noise in the signal in its latent space. Recently, EEG-based studies have utilized auto-encoders in an attempt to increase the classification accuracy. Promising results was obtained in the research by Yin and Zhang [35] as they used a stacked-denoising autoencoder to learn within and across sessions of EEG workload data. They achieved an average accuracy of 95.4 % and 87.4 % for classifying two levels of mental workloads within and across sessions, respectively. Another study by [36] showed a result with an accuracy of 98.99 % using auto-encoder and KNN classifiers for emotional arousal classification.

We hypothesize that by using the latent representation produces by auto encoders can classify mental workload for more than two levels and we will be able to distinguish these differences using the features extracted from an auto-encoder. In this study, we train a deep auto-encoder artificial neural network, with three commonly used EEG features; the brain load index (BLI), the power values of EEG frequency bands (alpha, beta and theta), and the relative gamma (RG). The encoded latent representations of these features, which is a projection of the actual features in a latent space, is then used for classification. To determine the best-performing feature we trained and tested both the EEG feature and their latent representations using seven different classifiers: support vector machines (SVMs) [37], the AdaBoost classifier [38], linear discriminant analyses [39], the ridge classifier [40], Random Forest [41], K-nearest neighbor Classifier and the Deep Belief Networks [42]. The results show that the classification accuracy was 83% using the SVM classifier with the power values of EEG frequency bands, and 91% with the latent representation of the same EEG’s power feature. This result indicates that the power of the EEG’s frequency band in a latent space could be the most suitable feature for classifying the different levels of lab-induced stress. The results suggest that the latent representation of these features, especially the power values, increased the classification accuracy by 8%. In this paper we also separately evaluate the classification performance for nurses and non-health professionals to understand their underlying differences in classification accuracies.

## II. Experiment and Methodology

### A. Participants and Instrumental Setup

Data was collected from 80 participants aged between 18 to 45 years old. Among the participants, there was 30 clinically active nurses and 50 non-health professionals. The participants was screened for medication use, smoking habits, alcohol intake and chronic disease/illness before the experiment. Any participant who did not qualify after the screening process was excluded. The experimentation protocol was approved by the University of Technology Sydney Human Research Ethics Committee (HREC), and all the participants provided informed written consent prior to the experiment. Brain activity was recorded with a 32-channel EEG cap using the NeuroScan EEG system, SynAmps 2 (Compumedics Limited, Victoria, Australia) at a sampling rate of 1000 Hz. The 32-EEG electrodes was placed according to the extended 10-20 international system [43], and the contact impedance was maintained below $5~k\Omega $.

### B. Experiment Design

The participants performed a modified version of the Trier Social Stress Test (TSST) [44] to elicit a controlled stress response. An EEG was recorded during the TSST, which involved 10-minute resting session, followed by a 10 minute preparation task, where participants was asked to prepare for a short speech. The preparation task was then followed by a 5 minute public speaking task, followed by 5 minutes of a difficult arithmetic task. The EEG recording obtained during the resting session was used as a baseline measurement.

## III. Data Processing

The EEG’s data processing was performed using EEGLAB [45], a toolbox in MATLAB (Mathworks Inc, USA). Raw EEG data was filtered using a 1 Hz high-pass and a 50 Hz low-pass infinite impulse response (IIR) filter. IIR is a recursive filter, which computes the current output by using current and previous inputs and also the previous outputs. By utilising previous input and previous output signals, IIR filters execute filter structure with feedback, this enables the filtering to work with fewer coefficients and thus work faster [46]. After filtering, the data was down-sampled to 250 Hz. Subsequently, an artifact subspace reconstruction (ASR) [47], [48] was applied to remove noisy channels and line noises. After the ASR, the data was re-referenced to a common average. An independent component analysis (ICA) [49] was then applied to decompose the data into independent components (ICs). The ICs related to eye movement, muscle activity; other noise was selected using a function in EEGLAB called IClabel [50] and ICs which had 90% likelihood of eye movements and muscle activity was removed. After the rejection of the bad components, dipole fitting [51], [52] was applied to the dataset in order to obtain the residual variance, and any component with a residual variance greater than 10% was also removed. After preprocessing the data, we found that 5 participants (3 belong to nurse group and 2 belong to non-health professional group) had unacceptable levels of noise in the data; therefore, data from these participants was excluded. Hence, subsequent analysis focused on the remaining 75 participants.

In the resultant data, a baseline correction was made by deducting the mean of the baseline data from the speech task, the preparation task, and the difficult arithmetic data. The EEG data of each task was divided into 1-second windows. An average value of alpha (7-13 Hz), beta (13-39 Hz) and theta (4-7 Hz) power values was computed for each window, which resulted in 600 data points for the baseline, 600 data points for the preparation task, 300 data points for the difficult arithmetic task and 300 data points for the speech task for each participant. Therefore, in total there was 600 x 75 data points for the baseline, 600 x 75 data points for the preparation task, 300 X 75 data points for the mental arithmetic task and 300 x 75 data points for speech task.

## IV. Feature Extraction Method

**FIGURE 1.**
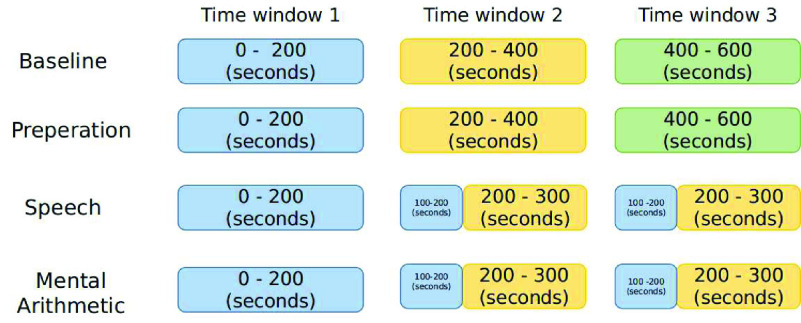
Division of data for different tasks. EEG recorded during baseline (resting session) and preparation task was divided into 3 time windows (time window 1 for first 200 seconds ≈ 200 data points, time window 2 for 200 to 400 seconds ≈ 200 data points, time window 3 for 400 to 600 seconds ≈ 200 data points). For speech and mental arithmetic tasks the first time window contains 0-200 seconds ≈ 200 data point, and time window 2 and time window 3 are the same which contain 100 to 300 seconds ≈ 200 data points.

Stress levels can change at different times during the task (e.g. beginning of the task, during the task, and at the end of the task). To understand which time window can be best classified, we divided the EEG data of each task into 3-time windows. As explained in section III, the baseline task contained 600 data points, which was divided into 1-200, 200-400 and 400-600 data points, and likewise for the preparation task. For the speech and mental arithmetic task, the first time window contains 1-200 data points, the second time window contains 100-300 data points, and the third time window contains 100-300 data points. Figure 1 shows the partitioning of the data per task. The time window 2 and 3 for speech and mental arithmetic are the same, however,for easy refereeing and consistency of the windows throughout the paper for the tasks, we used this notation.

### A. EEG Power Values at Central Positions: FZ, CZ and PZ Channels

**FIGURE 2.**
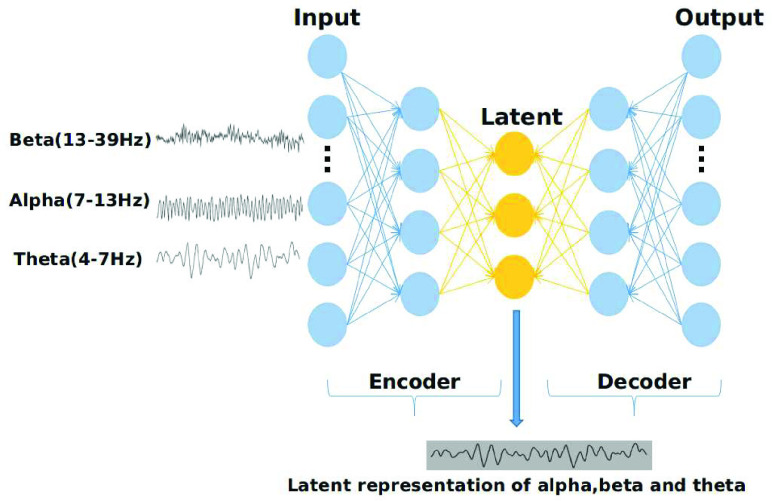
Auto encoder neural network, which receives alpha, beta and theta values.

The alpha (7-13 Hz), beta (13-39 Hz) and theta (4-7 Hz) power values for the Fz, Cz, and Pz channels was extracted using Welch’s method [53] using the EEGLab toolbox [45] for each time-window. Later, an auto-encoder of latent representations of size 50 was trained using each time-window, resulting in three trained auto-encoders. The architecture is shown in Figure 2.

### B. EEG Relative Gamma (RG) at Central Channels: FZ, CZ and PZ Channels

**FIGURE 3.**
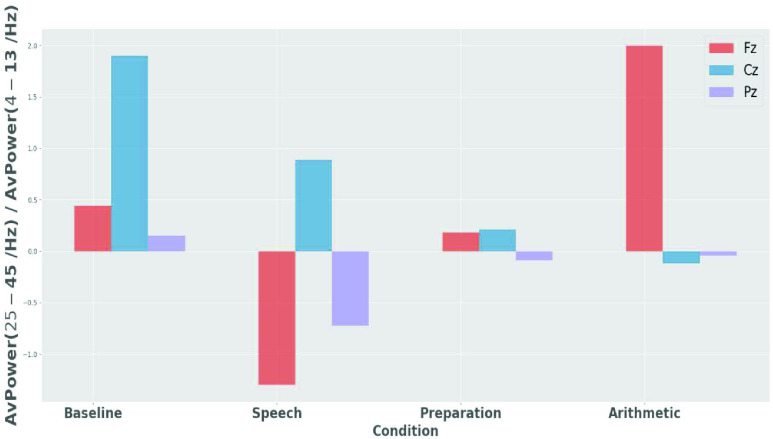
Mean RG for channel Fz, Cz and Pz across all participants for each condition.

The relative gamma is measured based on the complementarity of the fast and slow brain rhythms. The change in gamma oscillations in relation to the slow rhythms (4-23 Hz) play a complementary function to the fast rhythms [17]. The RG values 1 (as in equation 1) was computed from all the central channels (Fz, Cz and Pz) as features. The data was divided into three time-windows, similar to the power values. Figure 3 shows the mean RG values for each channel, each condition, and averaged across all participants.

\begin{equation*} \mathrm {RG}= AvPower(25-45\mathrm {Hz}) / AvPower(4-13 \mathrm {Hz}) \tag{1}\end{equation*}

### C. Brain Load Index (BLI)

**FIGURE 4.**
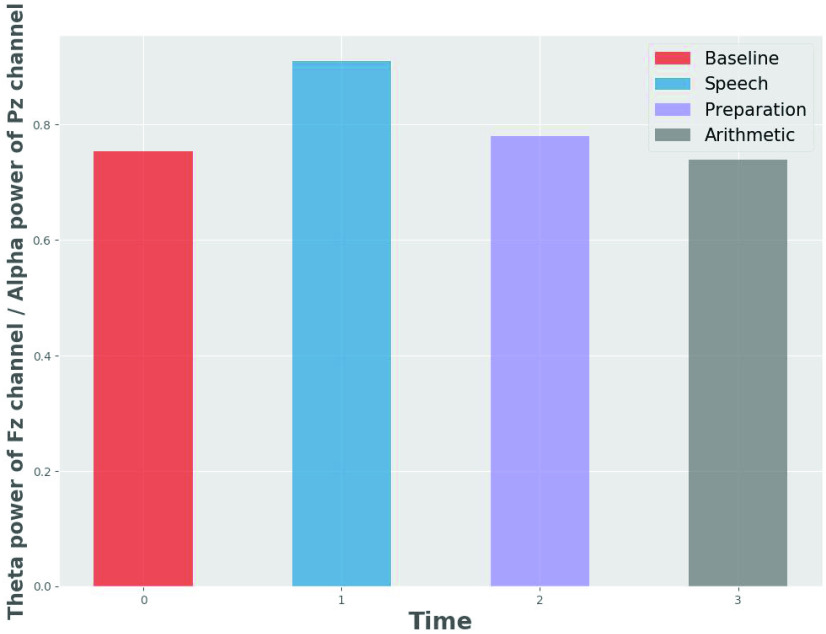
Mean BLI for channel Fz, Cz and Pz across all participants for each condition.

Studies on cognitive loads suggest that stress may increase the alpha activity in the right prefrontal cortex [21], [54]. The brain load index, which is the ratio of the frontal theta and the parietal alpha activities, is based on the theory that the increased cognitive stress increases the frontal theta activity and decreases the parietal alpha activity [55]. Thus, we used the brain load index (as in equation 2) as a feature. The data was divided into three time-windows, and each time-window was trained and tested by a 5-fold cross-validation using four classifiers. Fig 4 shows the mean BLI across all the participants for each condition.

\begin{equation*} \mathrm {BLI}= Theta(Fz)/ Alpha(Pz) \tag{2}\end{equation*}

### D. Autoencoder for Latent Representation

An auto-encoder is an unsupervised neural network which applies back propagation to make the output of the network similar to the input [44]. Given a signal $X$, an encoder network $Z = f(X)$ converts $X$ input to $Z$ latent representation, and decoder network $X^{prime} = g(Z)$ produces a reconstruction of the inputs $X$.

The weights of the neural network are learned by minimizing the following loss function equation 3.

\begin{equation*} L = \sqrt {1/N * \sum _{n=1}^{N}(x_{i}-x^{\prime }_{i})^{2}} \tag{3}\end{equation*}

where N is the total number of input points, $x_{i}$ is the $i_{th}$ input and $x^{\prime }_{i}$ is the $i_{th}$ node output.

By limiting the number of hidden units, more features in the data will be revealed. Figure 2 shows the structure of the auto-encoder. We trained an auto-encoder that was constituted by 50 hidden (latent) neurons. The root mean square error was used as a loss function, and the stochastic gradient descent algorithm was used as an optimizer. Each of the three features, namely, the power values, the RG and the brain load index, was used to train three different auto-encoders. Each of the three auto-encoders was trained separately. Later, the latent representations was extracted from these neural networks and used as features for the classification.

## V. Machine Learning Classifiers

We used four different classifiers to analyse the classification’s performance on both the original features and their latent representations. The following four classifiers was shown to be capable of classifying highly non-linear data [56]–[58].

### A. Support Vector Machine

A support vector machine (SVM) [37] performs classification by finding the hyper-plane that maximizes the boundary between multiple classes. The EEG data we used in this study was highly non-linear and was separable using an N-dimensional hyper-plane. However, the SVM used a non-linear kernel function to project the EEG data into a different space making it possible to perform the linear separation.

### B. AdaBoost

An AdaBoost [38] combines multiple classifiers (base estimators) to build strong classifiers. This study used 10 decision tree classifiers as base estimators. In each iteration, the AdaBoost trains its base estimators using randomly selected training subsets. It then assigns the weight to the trained base estimators in each iteration based on their accuracy. This process is iterated until all of the training data fits with good accuracy, or until it reaches the maximum number of estimators. In this study we used a maximum of 50 estimators.

### C. Linear Discriminant Analysis

A linear discriminant analysis (LDA) [39] is a dimensionality reduction technique which reduces the dimensions of the given data by removing the redundant features. An LDA transforms the features from a higher dimensional space to a lower dimensional space. This is achieved by maximizing the variance between the classes and minimizing the variance within the classes. An LDA estimates the probability that new data belongs to each class based on the mean and variance of the new data point. The performance of LDAs in this study also explains the separability of the distributions of multiclass EEG data.

### D. Ridge Classifier

Similar to the SVM, a ridge classifier [40] defines a hyperplane that can best separate the classes. However, the ridge parameter in this algorithm allows it to work on data that are not completely linearly separable. This study attempts to compare the performance of an SVM and a ridge classifier to understand if the learning of ridge parameters contributes to the classification.

### E. Random Forest Classifier

Random forest builds an ensemble of decision trees and combines the results of all the decision trees to get a more accurate prediction [41]. Each decision tree contains branches and nodes. At each node in the decision tree, a random set of features are regarded to decide the most beneficial split. Each decision tree is trained on each of the subsets. The final prediction is computed by averaging the prediction from all decision trees combined. In this study we used a Random forest classifier with 100 decision trees.

### F. K-Nearest Neighbors (KNN) Classifier

K-nearest neighbors (KNN) classify data based on the similarity measures (e.g., Euclidean distance) [59]. $k$ is the number of nearest neighbors that will be considered to predict the class of a new data point. For example, to classify a new test data point $x_{1}$, KNN algorithm first finds $k$ closest points to $x_{1}$ and then classify $x_{1}$ by preponderance of its $k$ neighbors. In this study, we used $k=5$ and minkowski [60] as a distance measure.

### G. Deep Belief Network (Deep BF)

Deep belief network [42] is a form of the deep neural network, which contains several layers of connected neurons. The training process is done in a greedy layer-wise fashion, where the weights are learned to abstract the hierarchical features from the data. Promising results were obtained when Deep BF networks were used for EEG classification [61], [62]. However, these existing studies either use EEG signals containing notable biomarkers such as P300 or use the Deep BF for binary-level classification. In this study, we also trained Deep BF networks to understand their ability to classify a multi-level stress task.

## VI. Performance Metrics

### A. T-Distributed Stochastic Neighbor Embedding

To explain the variance-covariance structure of the original features and the latent features we analysed the data using a T-distributed stochastic neighbor embedding (TSNE) [63]. A TSNE transforms similarities in the data to joint probabilities and minimizes the Kullback-Leibler [64] divergence between the joint probabilities of the low-dimensional embedding and the high-dimensional data. In this study we analyzed the TSNE by setting the perplexity to 10% for 5000 iterations with a learning rate of 10^3^.

## VII. Computational and Time Complexity

In this study we classified multi-level stress, using features extracted from AutoEncoder neural network and these features are then used for classification by Support Vector Machine (SVM). For training a Support Vector Machine classifier the time complexity is between $O(n^{2})$ and $O(n^{3})$, where $n$ is the number of training instances [65]. The minimum time complexity $O(n^{2})$, is possible when the classification boundary is explicit in the dataset. AutoEncoder is trained using the backpropagation algorithm, which involves forward propagation and backward propagation. Run-time complexity of a forward computation for layer $k$ with $n^{k}$ neurons of a neural network can be defined as $O(n^{k})$ [66]. In this study, we used an autoencoder neural network with 8 layers (4 layers for encoding and 5 layers for decoding). The time complexity for one feedforward pass of an AutoEncoder with 4 encoding layers is $O(ij + jk +kl)$, where $i, j, k and l$ are the number of neurons in each encoder layer and feedforward pas of 4 layer decoder is $O(ab + bc+ cd)$, where $a, b, c and d$ are the number of neurons in each decoder layer. the time complexity for a backward pass of an AutoEncoder with 4 encoder layers is $O(lk + kj + ji)$ and for 4 decoder layers is $O(dc + cb + ba)$. In this study we used 100 neurons in the input layer, followed by 50 neurons in each of the 3 encoder layers. The decoder contains 3 layers with 50 neurons each and 100 neurons in the last layer.

## VIII. Results

### A. Classification Results

Seven different classifiers was trained and tested in a 5-fold cross-validation method. Table 1 shows the classification accuracy of the support vector machine, the AdaBoost classifier, the LDA, the ridge classifiers, Random Forest, K-Nearest neighbor and Deep belief network. In this paper we present only the accuracy results of the above classifiers on the third time-window data (200-300 seconds data), which showed better performance compared to the first and second time-windows. Refer to the appendix for detailed results for each time-window. We also trained the four classifiers using raw amplitude of EEG data extracted from the Fz, Cz, and Pz channels. The classification accuracy was found to be 30%, and therefore was discarded for further analysis.

**TABLE 1:** Classification Accuracy of 7 Classifiers

Classifiers	EEG Bio-markers			Latent features		
	Power values	RG	BLI	latent power	latent RG	late nt BLI
SVM	0.83	0.23	0.46	0.91	0.24	0.80
AdaBoost	0.53	0.55	0.50	0.49	0.43	0.51
LDA	0.75	0.53	0.52	0.54	0.47	0.63
Ridge	0.51	0.45	0.54	0.49	0.45	0.63
Random Forest	0.81	0.55	0.65	0.87	0.49	0.77
KNN	0.85	0.46	0.6	0.89	0.45	0.67
Deep BF	0.13	0.18	0.16	0.23	0.18	0.18

**FIGURE 5.**
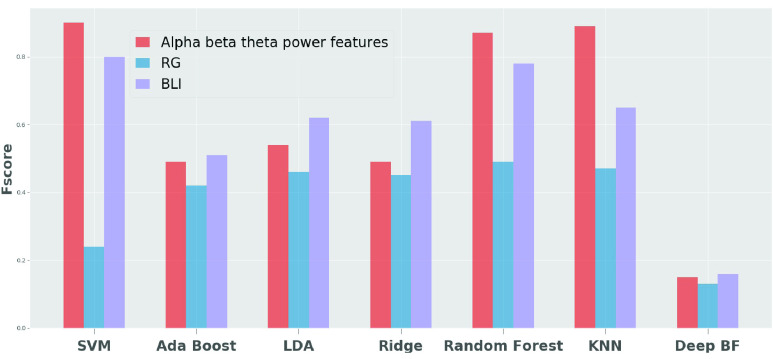
F-score for the classification using latent features.

**FIGURE 6.**
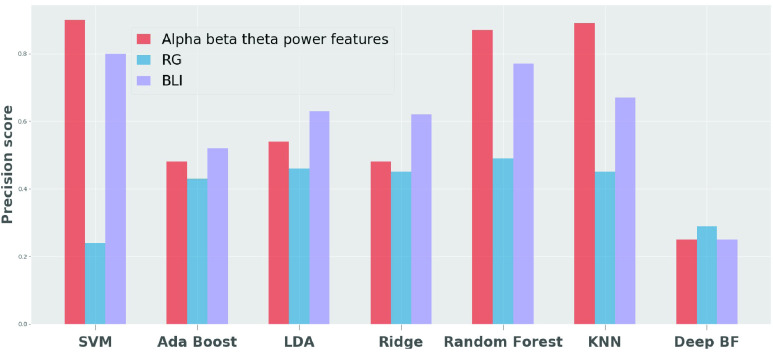
Precision score for the classification using latent features.

As shown in Table 1, the support vector machine algorithm outperformed the AdaBoost, LDA, ridge classifiers, Random Forest, K-Nearest neighbor and Deep belief network. The support vector machine, when trained on the alpha, beta, and theta power values, obtained a classification accuracy of 83%, and when trained using its corresponding latent features, the performance increased by 8%. The support vector machine also showed performance increases when trained on RG and BLI. However, the comparative power values showed significant differences in the classifications. Figure 5 and 6 shows the precision score and f-score obtained for 7 classifiers for the latent features.

### B. T-Distributed Stochastic Neighbor Embedding

**FIGURE 7.**
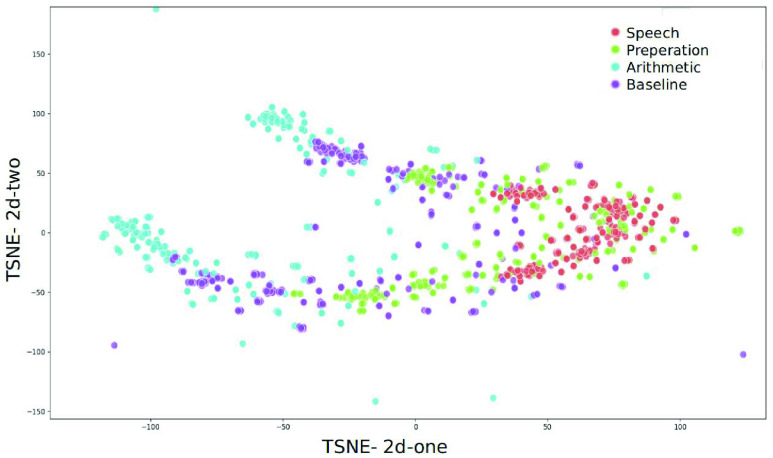
Alpha, beta and theta power values in TSNE 2 dimensions.

**FIGURE 8.**
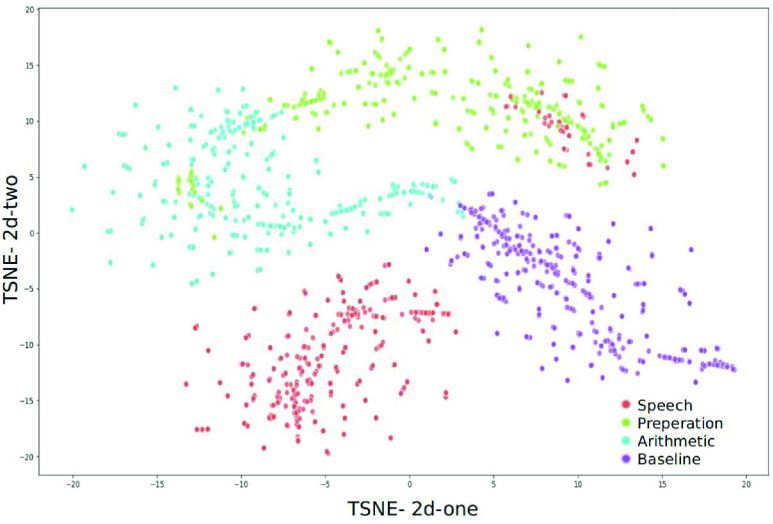
Latent representation of alpha, beta and theta in TSNE 2 dimensions.

To understand the local structural data differences between the feature and their latent features, we applied the TSNE algorithm on the best performing features, i.e. the alpha, beta, and theta power values and their latent features. Figure 7 and Figure 8 show the TSNE representations of the alpha, beta and theta power values and the latent features, respectively. This visualization explains the separability of the classes in to 2 kinds of features (original features and their latent features).

### C. Latent Representations for BLI, RG and the Power Values Features

Table 2 shows the mean and standard deviation of all the 6 features (power values, relative gamma (RG), brain load index (BLI) and their respective latent values).

**TABLE 2:** Mean and Standard Deviation of Latent Features

Latent features	Speech	Preparation	Arithmetic	Baseline
Power values Fz	−0.08 ± 4.14	−0.10 ± 4.11	−0.09 ± 6.21	−0.13 ± 7.18
Power values Cz	0.083 ± 8.50	0.01 ± 6.03	0.08 ± 6.20	0.048 ± 6.38
Power values Pz	−0.12 ± 4.32	−0.16 ± 4.27	−0.09 ± 4.37	−0.15 ± 4.24
RG	−0.087 ± 14.43	−0.013 ± 14.868	−0.179 ± 15.156	0.008 ± 12.249
BLI	0.037 ± 0.25	0.005 ± 0.279	−0.001 ± 0.49	−0.005 ± 0.348

## IX. Nurse and Non Health Professional Group

In order to understand the classification accuracy of different groups of participants, we analyzed the EEG data of 30 clinically active nurses. Table 3 shows the classification accuracies of the support vector machine, AdaBoost classifier, linear discriminant, the ridge classifiers, Random Forest, K-Nearest neighbor and Deep belief network. Table 4 shows the classification accuracies of 7 classifiers on the controller data (non-health professional group).

**TABLE 3:** Accuracy of 7 Different Classifiers, Trained on the Nurse Data to Classify Baseline, Arithmetic, Preparation and Speech Task

Classifiers	EEG Bio-markers			Latent features		
	Power values	RG	BLI	latent power	latent RG	latent BLI
SVM	0.86	0.31	0.34	0.75	0.42	0.807
AdaBoost	0.41	0.35	0.56	0.55	0.36	0.467
LDA	0.88	0.51	0.36	0.66	0.57	0.431
Ridge	0.79	0.45	0.42	0.644	0.54	0.36
Random Forest	0.71	0.48	0.78	0.78	0.48	0.82
KNN	0.81	0.44	0.61	0.87	0.45	0.61
Deep BF	0.52	0.16	0. 13	0.53	0.16	0.16

**TABLE 4:** Accuracy of 7 Different Classifiers, Trained on the Controller Data to Classify Baseline, Arithmetic, Preparation and Speech Task

Classifiers	EEG Bio-markers			Latent features		
	Power values	RG	BLI	latent power	latent RG	latent BLI
SVM	0.92	0.31	0.73	0.96	0.42	0.76
AdaBoost	0.57	0.35	0.49	0.74	0.36	0.63
LDA	0.71	0.51	0.36	0.75	0.57	0.49
Ridge	0.68	0.45	0.53	0.76	0.54	0.65
Random Forest	0.93	0.51	0.89	0.95	0.45	0.68
KNN	0.92	0.44	0.61	0.87	0.45	0.61
Deep BF	0.13	0.17	0.18	0.63	0.19	0.18

## X. Discussion

This study hypothesized that using the latent representations of bio-markers such the brain load index, the relative gamma and the power values of the EEG frequency bands (alpha, beta and theta) can improve the classification performance for 3 types of lab induced stress as compared to the traditional bio-markers. These bio-markers when projected in a latent space using a trained deep auto-encoder neural network, has created a better feature representation for classification. When tested these latent feature using four different classifiers, we observed that the latent features was able to classify the better in comparision to the actual bio-markers.

This research showed that the use of an auto-encoder allowed for prediction of changes across the averaged EEG central reference points. In this paper, the results obtained by combining the data from both control (non-nurse) participants, and nurses, in the experiment achieved an accuracy of 91 % when using the latent representation learned from the support vector machine. The accuracy increased to 91 % with the latent representations of the power values, in comparison to 83 % achieved with the actual power values. Disentangling the learning of feature extraction and classification, using our proposed framework has shown to increase the accuracy in classifying multi-level stress activity. The result of the SVM with the latent representation of BLI, also achieved a high accuracy of 80.7 %. These results suggest that the SVM with latent representation is a suitable and efficient classifier for addressing the changes of brain activity associated with various stress levels. Fig 6 shows the precision score when a latent representation is used for classification, and Fig 5 shows the f-score. The auto encoder neural network, when trained using an unsupervised algorithm, has been shown to extract the features of the power values, which resulted in improved classification performance. Studies [67]–[69] show that using the auto encoder neural network reduces the noise in the input data and extracts the essential features by projecting them into the latent space. These studies and the classification accuracy in this study suggest that the auto encoder removed the noise in the power values and extracted the features that increased the classification accuracy. he features extraction and classification algorithm are performed using only 3 central channels (Fz, Cz and Pz). This makes it feasible to use on a wearable EEG device. The proposed model can identify different stress-level conditions from the EEG data with high accuracy for both nurses and the control groups. To further enhance the accuracy, we will study the differences in brain dynamics under various stress-level conditions in a future study.

The classification of raw EEG data into 4 conditions (baseline, preparation, speech and arithmetic task) achieved an accuracy of 30%. In addition to artifacts, one major cause of this low accuracy could be the highly nonstationary and nonlinear dynamics of the recorded EEG data. Since the EEG data was recorded separately in stages (baseline, preparation, speech and arithmetic task), the issues of user variability, circadian variability, and task variability become more pronounced, and they induced shifts in the statistical properties in the data, leading to poor generalizations in the trained model. The proposed model uses features such as Brain Load index (BLI), Relative Gamma (RG) and power values (alpha, beta and theta of central channels) which are extract from 3 central channels (Cz, Fz and Pz). However, for some of the EEG datasets, it is essential to understand the features using EEG activity from all the channels. It still remains unexplored up to what degree the proposed architecture can learn to extract efficient features from all channel EEG activity.

Latent representation produced by auto-encoder has shown to extract the concealed bio-markers of stress. These latent representation has shown to provide features that are beneficial for further classification. By employing our proposed approach, various EEG datasets could be used to understand the feature representation. Additionally, using AutoEncoder with long-short term memory (LSTM) cells [70] can assist to understand the underlying temporal dynamical representation that can explain the variance in the EEG data. In the future, we aim to study the underlying temporal dynamics of multi-level stress from EEG data using AutoEncoder with LSTM cells. The proposed model can also be investigated in the direction of detecting stress level in real-time, which might be a potential application for therapeutic strategy.

## XI. Conclusion

In summary, this paper demonstrated that the most suitable feature to detect stress are the power values of the alpha, theta and beta frequencies. For identifying the four conditions, an average classification accuracy of 83 % was achieved using the SVM with a power value of 91 % with the latent representation of the power value. Furthermore, the results also demonstrated the viability of an autoencoder for increasing the classification accuracy, since the latent representations of the features might encapsulate the features.

## References

[1] J. Campbell and U. Ehlert, “Acute psychosocial stress: Does the emotional stress response correspond with physiological responses?,” Psychoneuroendocrinology, vol. 37, no. 8, pp. 1111–1134, Aug. 2012. 22260938 10.1016/j.psyneuen.2011.12.010

[2] D. A. Weinberger, G. E. Schwartz, and R. J. Davidson, “Low-anxious, high-anxious, and repressive coping styles: Psychometric patterns and behavioral and physiological responses to stress,” J. Abnormal Psychol., vol. 88, no. 4, p. 369, 1979. 10.1037//0021-843x.88.4.369 479459

[3] G. Durantin, J.-F. Gagnon, S. Tremblay, and F. Dehais, “Using near infrared spectroscopy and heart rate variability to detect mental overload,” Behav. Brain Res., vol. 259, pp. 16–23, Feb. 2014. 24184083 10.1016/j.bbr.2013.10.042

[4] C. Kirschbaum, K.-M. Pirke, and D. H. Hellhammer, “The ‘trier social stress test’—A tool for investigating psychobiological stress responses in a laboratory setting,” Neuropsychobiology, vol. 28, nos. 1–2, pp. 76–81, 1993. 8255414 10.1159/000119004

[5] G. Specchia, “Mental arithmetic stress testing in patients with coronary artery disease,” Amer. Heart J., vol. 108, no. 1, pp. 56–63, Jul. 1984. 6731283 10.1016/0002-8703(84)90544-1

[6] J. F. Thayer, F. Åhs, M. Fredrikson, J. J. Sollers, and T. D. Wager, “A meta-analysis of heart rate variability and neuroimaging studies: Implications for heart rate variability as a marker of stress and health,” Neurosci. Biobehav. Rev., vol. 36, no. 2, pp. 747–756, Feb. 2012. 22178086 10.1016/j.neubiorev.2011.11.009

[7] Y. Shi, N. Ruiz, R. Taib, E. Choi, and F. Chen, “Galvanic skin response (GSR) as an index of cognitive load,” in Proc. CHI Extended Abstr. Hum. Factors Comput. Syst. (CHI), 2007, pp. 2651–2656.

[8] S. Cohen, R. C. Kessler, and L. U. Gordon, Measuring Stress: A Guide for Health and Social Scientists. London, U.K.: Oxford Univ. Press, 1997.

[9] T. Hwang, M. Kim, S. Hong, and K. S. Park, “Driver drowsiness detection using the in-ear EEG,” in Proc. 38th Annu. Int. Conf. IEEE Eng. Med. Biol. Soc. (EMBC), Aug. 2016, pp. 4646–4649. 10.1109/EMBC.2016.7591763 28269310

[10] Y. N. Singh, S. K. Singh, and A. K. Ray, “Bioelectrical signals as emerging biometrics: Issues and challenges,” ISRN Signal Process., vol. 2012, pp. 1–13, Jul. 2012.

[11] S. Park, C.-H. Han, and C.-H. Im, “Design of wearable EEG devices specialized for passive brain–computer interface applications,” Sensors, vol. 20, no. 16, p. 4572, Aug. 2020. 10.3390/s20164572 PMC7472161 32824011

[12] A. Casson, D. Yates, S. Smith, J. Duncan, and E. Rodriguez-Villegas, “Wearable electroencephalography,” IEEE Eng. Med. Biol. Mag., vol. 29, no. 3, pp. 44–56, 5 2010. 10.1109/MEMB.2010.936545 20659857

[13] M. Alvarado-González, E. Garduño, E. Bribiesca, O. Yáñez-Suárez, and V. Medina-Bañuelos, “P300 detection based on EEG shape features,” Comput. Math. Methods Med., vol. 2016, pp. 1–14, Jan. 2016. 10.1155/2016/2029791 PMC4736976 26881010

[14] M. Phothisonothai, “An investigation of using SSVEP for EEG-based user authentication system,” in Proc. Asia–Pacific Signal Inf. Process. Assoc. Annu. Summit Conf. (APSIPA), Dec. 2015, pp. 923–926.

[15] J. Minguillon, M. A. Lopez-Gordo, and F. Pelayo, “Stress assessment by prefrontal relative gamma,” Frontiers Comput. Neurosci., vol. 10, p. 101, Sep. 2016. 10.3389/fncom.2016.00101 PMC5031688 27713698

[16] S. R. Steinhubl, “Cardiovascular and nervous system changes during meditation,” Frontiers Hum. Neurosci., vol. 9, p. 145, Mar. 2015. 10.3389/fnhum.2015.00145 PMC4364161 25852526

[17] A. Lutz, L. L. Greischar, N. B. Rawlings, M. Ricard, and R. J. Davidson, “Long-term meditators self-induce high-amplitude gamma synchrony during mental practice,” Proc. Nat. Acad. Sci. USA, vol. 101, no. 46, pp. 16369–16373, Nov. 2004. 15534199 10.1073/pnas.0407401101 PMC526201

[18] J. Minguillon, E. Perez, M. Lopez-Gordo, F. Pelayo, and M. Sanchez-Carrion, “Portable system for real-time detection of stress level,” Sensors, vol. 18, no. 8, p. 2504, Aug. 2018. 10.3390/s18082504 PMC6111320 30071643

[19] A. Holm, K. Lukander, J. Korpela, M. Sallinen, and K. M. I. Müller, “Estimating brain load from the EEG,” Sci. World J., vol. 9, pp. 639–651, Jun. 2009. 10.1100/tsw.2009.83 PMC5823228 19618092

[20] S. Tiinanen, A. Mättä, M. Silfverhuth, K. Suominen, E. Jansson-Verkasalo, and T. Seppänen, “HRV and EEG based indicators of stress in children with asperger syndrome in audio-visual stimulus test,” in Proc. Annu. Int. Conf. IEEE Eng. Med. Biol. Soc., Aug. 2011, pp. 2021–2024. 10.1109/IEMBS.2011.6090371 22254732

[21] R. S. Lewis, N. Y. Weekes, and T. H. Wang, “The effect of a naturalistic stressor on frontal EEG asymmetry, stress, and health,” Biol. Psychol., vol. 75, no. 3, pp. 239–247, Jul. 2007. 17512106 10.1016/j.biopsycho.2007.03.004

[22] V. Knott, C. Mahoney, S. Kennedy, and K. Evans, “EEG power, frequency, asymmetry and coherence in male depression,” Psychiatry Res. Neuroimag., vol. 106, no. 2, pp. 123–140, Apr. 2001. 10.1016/s0925-4927(00)00080-9 11306251

[23] G. Jun and K. G. Smitha, “EEG based stress level identification,” in Proc. IEEE Int. Conf. Syst., Man, Cybern. (SMC), Oct. 2016, pp. 003270–003274.

[24] M. S. Kalas and B. F. Momin, “Stress detection and reduction using EEG signals,” in Proc. Int. Conf. Electr., Electron., Optim. Techn. (ICEEOT), Mar. 2016, pp. 471–475.

[25] R. Khosrowabadi, C. Quek, K. K. Ang, S. W. Tung, and M. Heijnen, “A brain-computer interface for classifying EEG correlates of chronic mental stress,” in Proc. Int. Joint Conf. Neural Netw., Jul. 2011, pp. 757–762.

[26] S. A. Hosseini and M. A. Khalilzadeh, “Emotional stress recognition system using EEG and psychophysiological signals: Using new labelling process of EEG signals in emotional stress state,” in Proc. Int. Conf. Biomed. Eng. Comput. Sci., Apr. 2010, pp. 1–6.

[27] R. Sharma, P. Sircar, and R. B. Pachori, “Automated seizure classification using deep neural network based on autoencoder,” in Handbook of Research on Advancements of Artificial Intelligence in Healthcare Engineering. Hershey, PA, USA: IGI Global, 2020, pp. 1–19.

[28] R. Sharma, R. B. Pachori, and P. Sircar, “Seizures classification based on higher order statistics and deep neural network,” Biomed. Signal Process. Control, vol. 59, 5 2020, Art. no. 101921.

[29] S. R. Nayak, D. R. Nayak, U. Sinha, V. Arora, and R. B. Pachori, “Application of deep learning techniques for detection of COVID-19 cases using chest X-ray images: A comprehensive study,” Biomed. Signal Process. Control, vol. 64, Feb. 2021, Art. no. 102365. 10.1016/j.bspc.2020.102365 PMC7674150 33230398

[30] S. Madhavan, R. K. Tripathy, and R. B. Pachori, “Time-frequency domain deep convolutional neural network for the classification of focal and non-focal EEG signals,” IEEE Sensors J., vol. 20, no. 6, pp. 3078–3086, Mar. 2019.

[31] P. K. Chaudhary and R. B. Pachori, “Automatic diagnosis of COVID-19 and pneumonia using FBD method,” in Proc. IEEE Int. Conf. Bioinf. Biomed. (BIBM), Dec. 2020, pp. 2257–2263.

[32] W. Sun, S. Shao, R. Zhao, R. Yan, X. Zhang, and X. Chen, “A sparse auto-encoder-based deep neural network approach for induction motor faults classification,” Measurement, vol. 89, pp. 171–178, Jul. 2016.

[33] M. Chen, X. Shi, Y. Zhang, D. Wu, and M. Guizani, “Deep features learning for medical image analysis with convolutional autoencoder neural network,” IEEE Trans. Big Data, early access, Jun. 20, 2017, doi: 10.1109/TBDATA.2017.2717439.

[34] D. R. Nayak, R. Dash, B. Majhi, R. B. Pachori, and Y. Zhang, “A deep stacked random vector functional link network autoencoder for diagnosis of brain abnormalities and breast cancer,” Biomed. Signal Process. Control, vol. 58, Apr. 2020, Art. no. 101860.

[35] Z. Yin and J. Zhang, “Cross-session classification of mental workload levels using EEG and an adaptive deep learning model,” Biomed. Signal Process. Control, vol. 33, pp. 30–47, Mar. 2017.

[36] D. Ayata, Y. Yaslan, and M. Kamasak, “Multi channel brain EEG signals based emotional arousal classification with unsupervised feature learning using autoencoders,” in Proc. 25th Signal Process. Commun. Appl. Conf. (SIU), May 2017, pp. 1–4.

[37] M. A. Hearst, S. T. Dumais, E. Osuna, J. Platt, and B. Scholkopf, “Support vector machines,” IEEE Intell. Syst. Appl., vol. 13, no. 4, pp. 18–28, Jul. 1998, doi: 10.1109/5254.708428.

[38] Y. Freund, R. Schapire, and N. Abe, “A short introduction to boosting,” J.-Jpn. Soc. Artif. Intell., vol. 14, nos. 771–780, p. 1612, 1999.

[39] P. Xanthopoulos, P. M. Pardalos, and T. B. Trafalis, “Linear discriminant analysis,” in Robust Data Mining. Springer, 2013, pp. 27–33.

[40] M. Grüning and S. Kropf, “A ridge classification method for high-dimensional observations,” in From Data and Information Analysis to Knowledge Engineering, M. Spiliopoulou, R. Kruse, C. Borgelt, A. Nürnberger, and W. Gaul, Eds. Berlin, Germany: Springer, 2006, pp. 684–691.

[41] L. Breiman, “Random forests,” Mach. Learn., vol. 45, no. 1, pp. 5–32, 2001.

[42] G. E. Hinton, “Deep belief nets,” Tech. Rep., 2010.

[43] R. W. Homan, J. Herman, and P. Purdy, “Cerebral location of international 10–20 system electrode placement,” Electroencephalogr. Clin. Neurophysiol., vol. 66, no. 4, pp. 376–382, Apr. 1987. 2435517 10.1016/0013-4694(87)90206-9

[44] D. H. Ballard, “Modular learning in neural networks,” in Proc. AAAI, 1987, pp. 279–284.

[45] A. Delorme and S. Makeig, “EEGLAB: An open source toolbox for analysis of single-trial EEG dynamics including independent component analysis,” J. Neurosci. Methods, vol. 134, no. 1, pp. 9–21, Mar. 2004. 15102499 10.1016/j.jneumeth.2003.10.009

[46] L. Litwin, “FIR and IIR digital filters,” IEEE Potentials, vol. 19, no. 4, pp. 28–31, 2000.

[47] C. Kothe, “The artifact subspace reconstruction method,” Tech. Rep., 2013. Accessed: Jul. 17, 2017.

[48] C. A. E. Kothe and T.-P. Jung, “Artifact removal techniques with signal reconstruction,” U.S. Patent 14 895 440, Apr. 28, 2016.

[49] S. Makeig, A. J. Bell, T.-P. Jung, and T. J. Sejnowski, “Independent component analysis of electroencephalographic data,” in Proc. Adv. Neural Inf. Process. Syst., 1996, pp. 145–151.

[50] L. Pion-Tonachini, K. Kreutz-Delgado, and S. Makeig, “ICLabel: An automated electroencephalographic independent component classifier, dataset, and website,” NeuroImage, vol. 198, pp. 181–197, Sep. 2019. 31103785 10.1016/j.neuroimage.2019.05.026 PMC6592775

[51] C. C. Wood, “Application of dipole localization methods to source identification of human evoked potentials,” Ann. New York Acad. Sci., vol. 388, no. 1, pp. 139–155, 1982. 6953865 10.1111/j.1749-6632.1982.tb50789.x

[52] R. Oostenveld, P. Fries, E. Maris, and J.-M. Schoffelen, “FieldTrip: Open source software for advanced analysis of MEG, EEG, and invasive electrophysiological data,” Comput. Intell. Neurosci., vol. 2011, pp. 1–9, Oct. 2011. 21253357 10.1155/2011/156869 PMC3021840

[53] P. Welch, “The use of fast Fourier transform for the estimation of power spectra: A method based on time averaging over short, modified periodograms,” IEEE Trans. Audio Electroacoust., vol. AU-15, no. 2, pp. 70–73, Jun. 1967.

[54] R. Davidson, “Frontal versus perietal EEG asymmetry during positive and negative affect,” Psychophysiology, vol. 16, no. 2, pp. 202–203, 1979.

[55] P. Sauseng, W. Klimesch, M. Schabus, and M. Doppelmayr, “Fronto-parietal EEG coherence in theta and upper alpha reflect central executive functions of working memory,” Int. J. Psychophysiol., vol. 57, no. 2, pp. 97–103, Aug. 2005. 15967528 10.1016/j.ijpsycho.2005.03.018

[56] H. Shimodaira, K.-I. Noma, M. Nakai, and S. Sagayama, “Dynamic time-alignment kernel in support vector machine,” in Proc. Adv. Neural Inf. Process. Syst., 2002, pp. 921–928.

[57] Q. Zhao and C. Koch, “Learning visual saliency by combining feature maps in a nonlinear manner using AdaBoost,” J. Vis., vol. 12, no. 6, p. 22, 2012. 10.1167/12.6.22 22707429

[58] D. W. Marquaridt, “Generalized inverses, ridge regression, biased linear estimation, and nonlinear estimation,” Technometrics, vol. 12, no. 3, pp. 591–612, Aug. 1970.

[59] H. U. Amin, W. Mumtaz, A. R. Subhani, M. N. M. Saad, and A. S. Malik, “Classification of EEG signals based on pattern recognition approach,” Frontiers Comput. Neurosci., vol. 11, p. 103, Nov. 2017. 10.3389/fncom.2017.00103 PMC5702353 29209190

[60] A. C. Thompson and A. C. Thompson, Minkowski Geometry. Cambridge, U.K.: Cambridge Univ. Press, 1996.

[61] S. A. Cortez, C. Flores, and J. Andreu-Perez, “Single-trial P300 classification using deep belief networks for a BCI system,” in Proc. IEEE XXVII Int. Conf. Electron., Electr. Eng. Comput. (INTERCON), Sep. 2020, pp. 1–4.

[62] S. A. Cortez, C. Flores, and J. Andreu-Perez, “Improving speller BCI performance using a cluster-based under-sampling method,” in Proc. IEEE Symp. Ser. Comput. Intell. (SSCI), Dec. 2020, pp. 576–581.

[63] L. van der Maaten and G. Hinton, “Visualizing data using t-SNE,” J. Mach. Learn. Res., vol. 9, pp. 2579–2605, Nov. 2008.

[64] S. Kullback and R. A. Leibler, “On information and sufficiency,” Ann. Math. Statist., vol. 22, no. 1, pp. 79–86, 1951.

[65] L. Bottou and C.-J. Lin, “Support vector machine solvers,” Large Scale Kernel Mach., vol. 3, no. 1, pp. 301–320, 2007.

[66] P. Orponen, “Computational complexity of neural networks: A survey,” Nordic J. Comput., pp. 1–7, 1994.

[67] L. Wu and S. Picek, “Remove some noise: On pre-processing of side-channel measurements with autoencoders,” IACR Cryptol. ePrint Arch., vol. 2019, p. 1474, 2019.

[68] X. Yu and F. Porikli, “Hallucinating very low-resolution unaligned and noisy face images by transformative discriminative autoencoders,” in Proc. IEEE Conf. Comput. Vis. Pattern Recognit., Jul. 2017, pp. 3760–3768.

[69] G. Eraslan, L. M. Simon, M. Mircea, N. S. Mueller, and F. J. Theis, “Single-cell RNA-seq denoising using a deep count autoencoder,” Nature Commun., vol. 10, no. 1, pp. 1–14, Dec. 2019. 30674886 10.1038/s41467-018-07931-2 PMC6344535

[70] A. Sagheer and M. Kotb, “Unsupervised pre-training of a deep LSTM-based stacked autoencoder for multivariate time series forecasting problems,” Sci. Rep., vol. 9, no. 1, pp. 1–16, Dec. 2019. 31836728 10.1038/s41598-019-55320-6 PMC6911101

